# Supplementary figures and images for: An Efficient and Cost-Effective Approach to Generate Functional Human Inducible Pluripotent Stem Cell-Derived Astrocytes
Source: Cells. 2023 Sep 26;12(19):2357. doi: 10.3390/cells12192357 (PMC10571578; doi:10.3390/cells12192357)

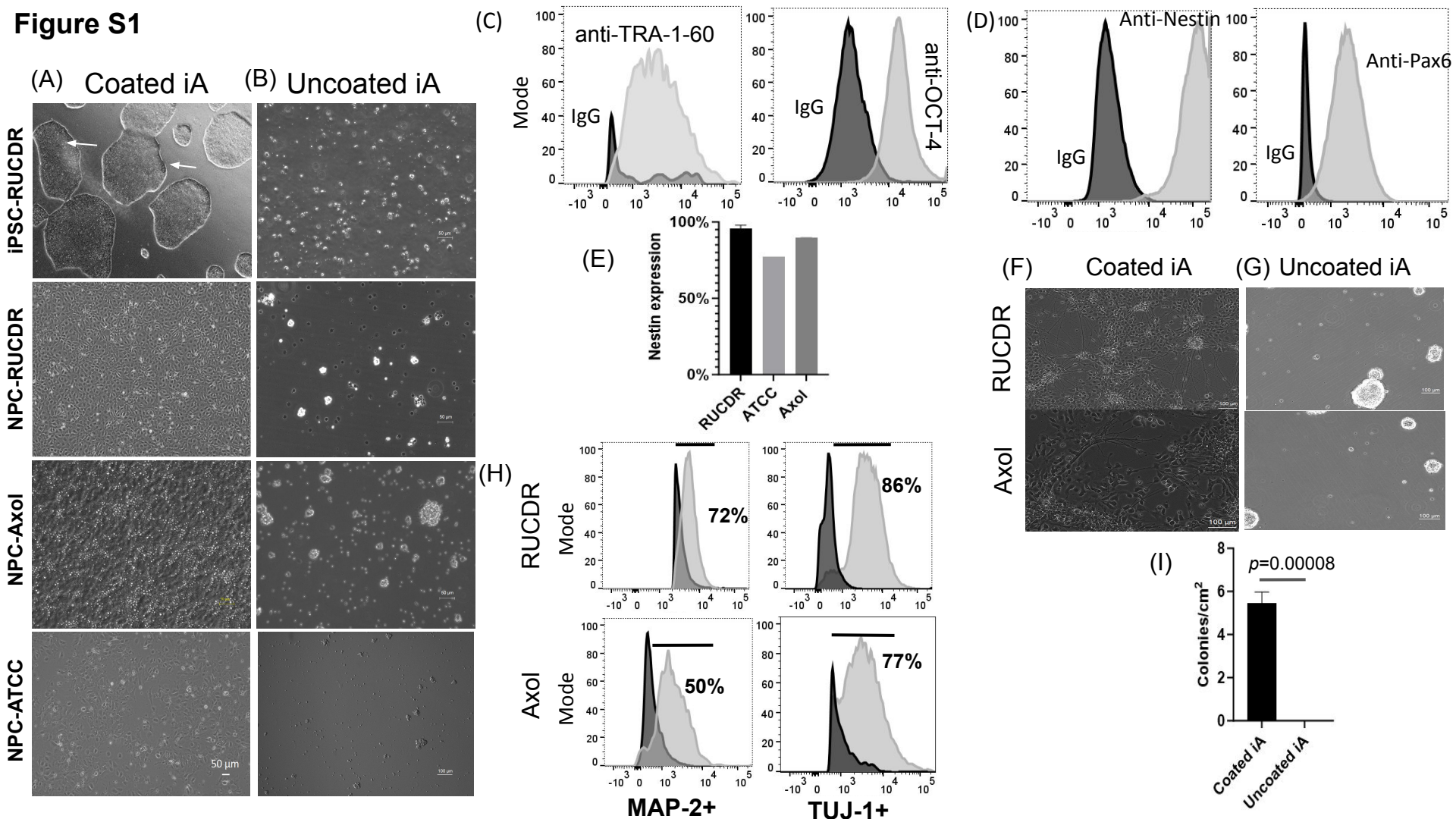

**Figure S2**

(A) Axol-iAS

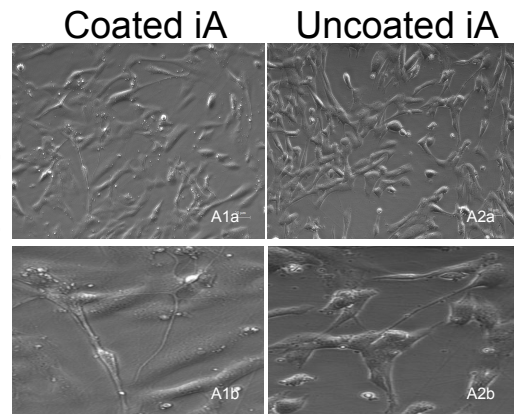

(B) ATCC-iAS

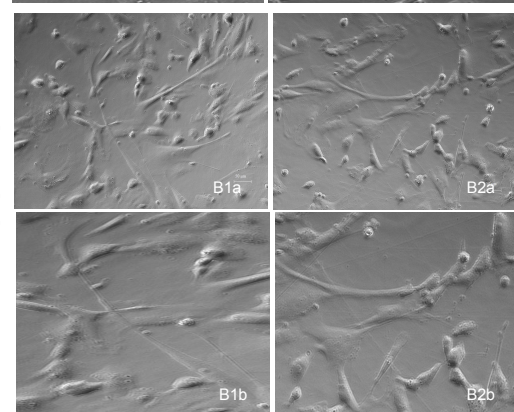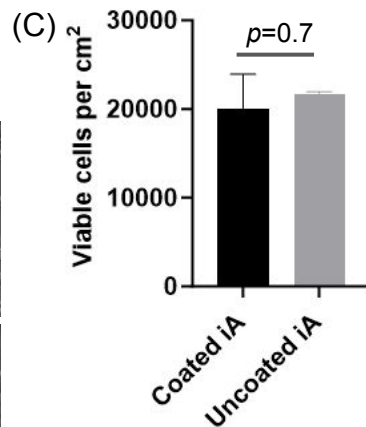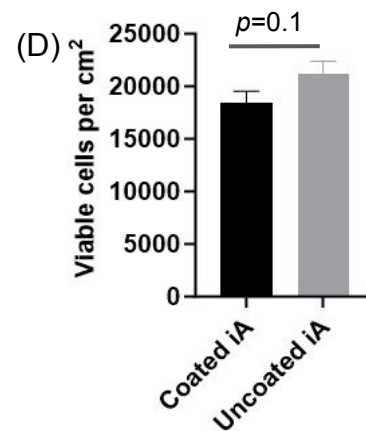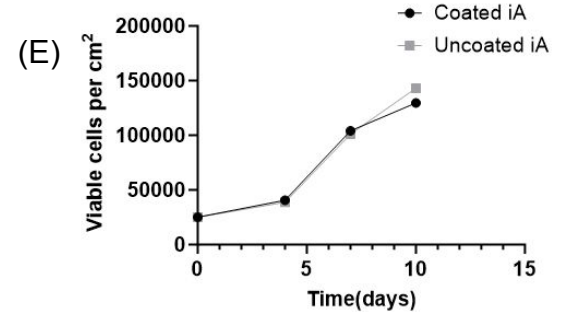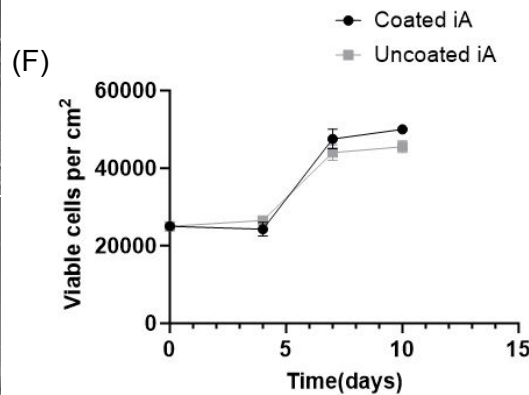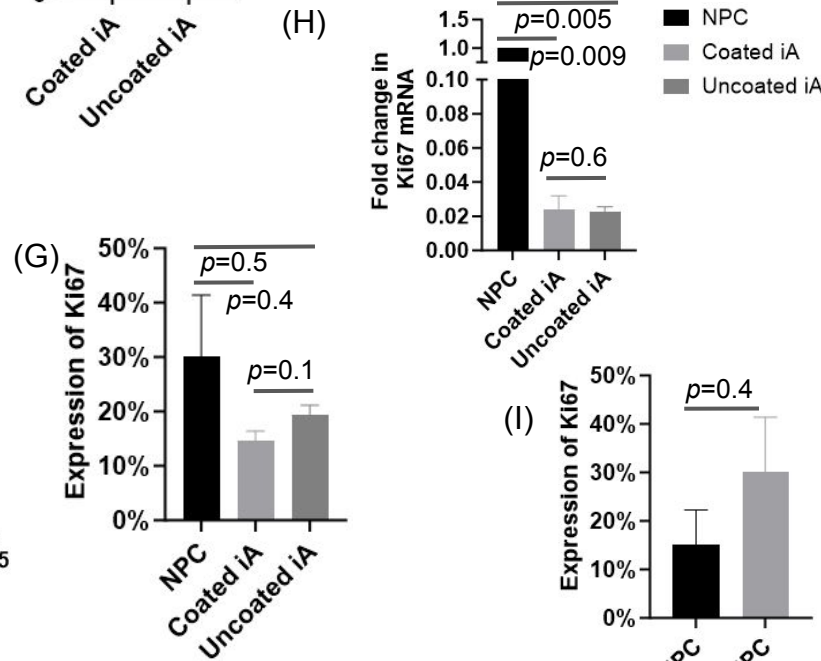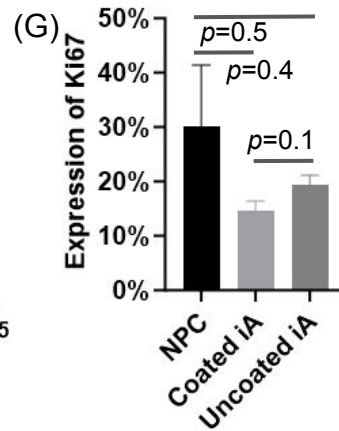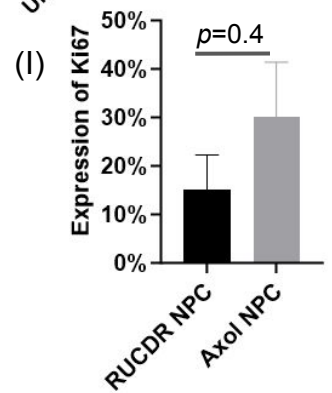

# Supplementary Figure S3

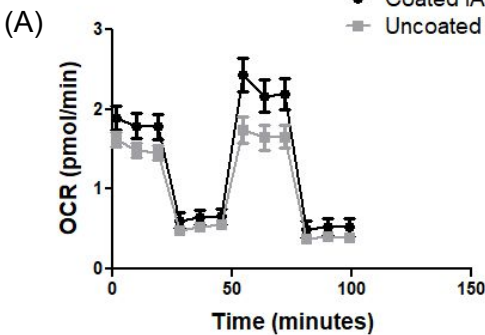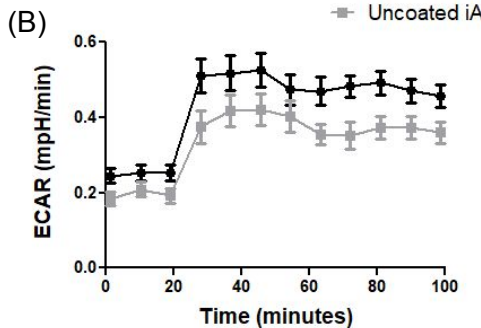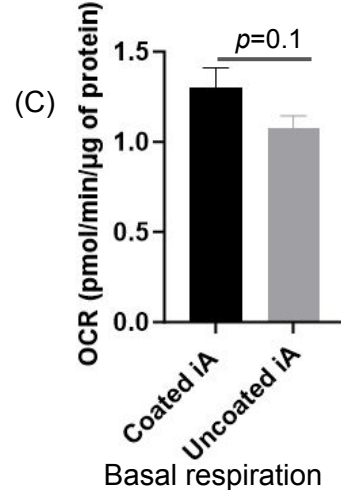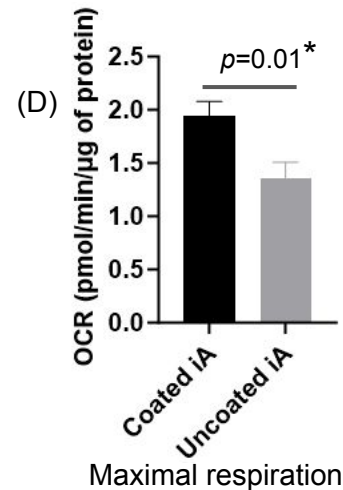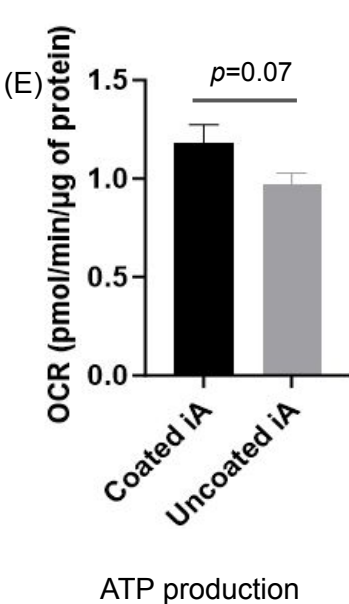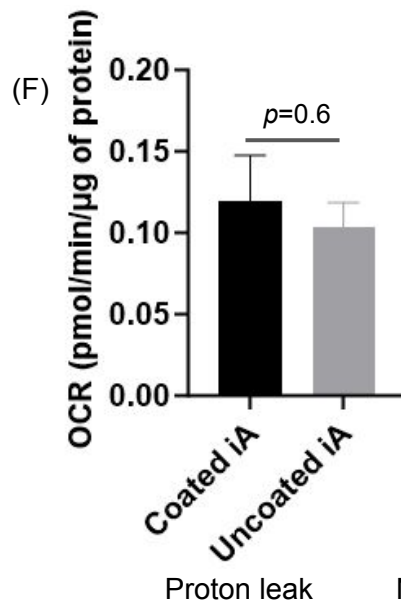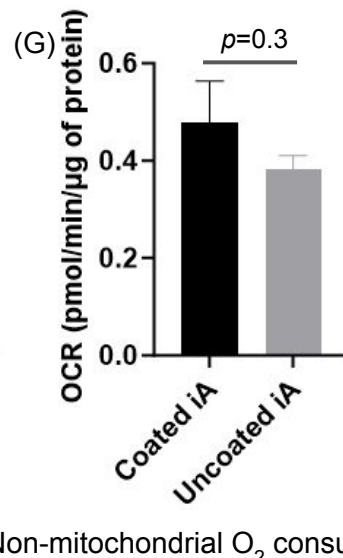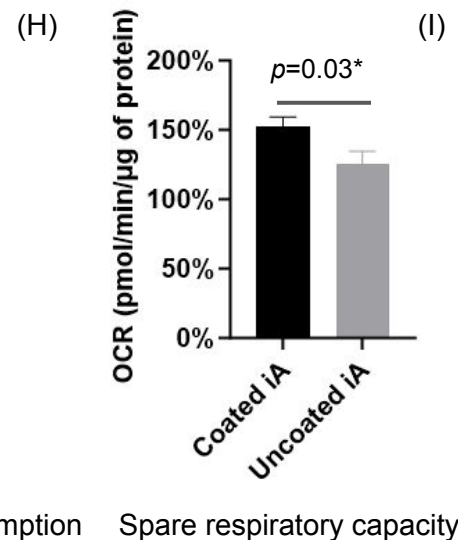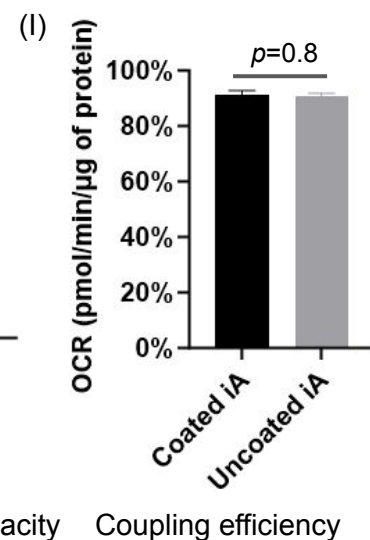

Supplement: Supplementary file 1 [file cells-12-02357-s001.zip › cells-2568536-supplementary.pdf]
